# Hepatocyte-targeted Bap1 reduction in the liver primes an inflammatory transcriptional response

**DOI:** 10.1093/g3journal/jkag047

**Published:** 2026-02-19

**Authors:** William C Nenad, Peyton C Kuhlers, Ian R Sturgill, Irene Biju, Max Bucklan, Lindsey Hernandez, Lee-Ching Zhu, Katherine A Hoadley, Jesse R Raab

**Affiliations:** Bioinformatics and Computational Biology Curriculum, Department of Genetics, The University of North Carolina at Chapel Hill, Chapel Hill, NC 27599, United States; Department of Genetics, The University of North Carolina at Chapel Hill, Chapel Hill, NC 27599, United States; Lineberger Comprehensive Cancer Center, The University of North Carolina at Chapel Hill, Chapel Hill, NC 27599, United States; Bioinformatics and Computational Biology Curriculum, Department of Genetics, The University of North Carolina at Chapel Hill, Chapel Hill, NC 27599, United States; Lineberger Comprehensive Cancer Center, The University of North Carolina at Chapel Hill, Chapel Hill, NC 27599, United States; Cancer Science Institute of Singapore, Center for Translational Medicine, Singapore 117599; Department of Genetics, The University of North Carolina at Chapel Hill, Chapel Hill, NC 27599, United States; Department of Genetics, The University of North Carolina at Chapel Hill, Chapel Hill, NC 27599, United States; Lineberger Comprehensive Cancer Center, The University of North Carolina at Chapel Hill, Chapel Hill, NC 27599, United States; Department of Pathology and Laboratory Medicine, The University of North Carolina at Chapel Hill, Chapel Hill, NC, 27599, United States; Department of Genetics, The University of North Carolina at Chapel Hill, Chapel Hill, NC 27599, United States; Lineberger Comprehensive Cancer Center, The University of North Carolina at Chapel Hill, Chapel Hill, NC 27599, United States; Department of Genetics, The University of North Carolina at Chapel Hill, Chapel Hill, NC 27599, United States; Lineberger Comprehensive Cancer Center, The University of North Carolina at Chapel Hill, Chapel Hill, NC 27599, United States

**Keywords:** BRCA1-associated protein 1, chemokine, cytokine, liver damage response, immune recruitment, Kupffer cells, fatty acid metabolism, spatial transcriptomics

## Abstract

BRCA1-associated protein 1 (BAP1) is a deubiquitinase, frequently altered in cancers including hepatocellular carcinoma and cholangiocarcinoma. While Bap1 has been shown to play key roles in metabolism, maintenance of tissue homeostasis, and immune cell development, little is known about its normal functions in the liver in vivo. Using AAV8-mediated CRISPR/CAS9 genome editing, we generated a mouse hepatocyte-specific model of Bap1 knockout to define the changes that occur in liver biology in an in vivo system and characterize how loss of Bap1 alters the liver's response to injury. Single-cell resolution spatial transcriptomics were performed in conjunction with immunohistochemistry to analyze cell-type composition and immune cell recruitment changes. Bulk RNA-sequencing was performed to further assess the impact of Bap1 loss on transcription. Hepatocyte-specific depletion of Bap1-induced transcriptional changes shared with acute injury. We observed a strong dysregulation of inflammatory pathways associated with Bap1 loss. Moreover, the transcriptional response of Bap1 depletion in hepatocytes to damage was markedly different than in control liver, with Bap1-depleted livers showing a decreased hepatocyte identity based on gene expression. Spatial transcriptomics and quantitative texture analysis of immunohistochemistry revealed an altered immune environment prior to damage and an impaired recruitment of immune cells in Bap1-depleted livers after damage. Our data suggest Bap1 is a critical modulator in the liver's immune cell response and its loss leads to an inflammatory environment prior to damage and disrupts the recruitment immune cells. Our quantitative spatial analysis highlights the power of such approaches to characterize the spatial distribution of different cell types in a tissue.

## Introduction

Bap1 is a tumor suppressor gene encoding a deubiquitinase whose main function is to remove ubiquitin from Histone H2A at Lysine 119 (H2AK119ub). H2AK119ub is deposited by Polycomb repressive complex 1 (PRC1), making BAP1 a critical brake on transcriptional repression. Chromatin dynamics play a critical role in acute and chronic injury responses as well as liver cancer (Hodges et al. 2016; Flavahan et al. 2017; Zhao et al. 2021; Wang et al. 2025). H2AK119ub serves as a docking site for Polycomb repressive complex 2 (PRC2), where PRC1/2 dynamics have been shown to control hepatocyte maturation and fibrogenesis (Blackledge et al. 2014; Grindheim et al. 2019; Yan et al. 2021). BAP1 inactivation is frequently observed in cholangiocarcinoma and a subset of hepatocellular carcinomas (Cancer Genome Atlas Research Network 2017; Farshidfar et al. 2017; Sturgill et al. 2024). BAP1 loss in a hepatocellular carcinoma cohort from The Cancer Genome Atlas (TCGA) molecularly resembled cholangiocarcinoma, suggesting that BAP1 may regulate cellular plasticity in the liver (Damrauer et al. 2021).

Bap1 is a key regulator in the liver, contributing to the maintenance of tissue homeostasis, normal liver function, and metabolism (Baughman et al. 2016). Artegiani et al. (2019) generated a human liver organoid model of BAP1 knockout and showed that BAP1 loss resulted in dysregulation of the epithelial cell state and identity. BAP1 plays a central role in immune cell development and responses through its ubiquitination activity in B-cell, T-cell, and macrophage development (Liang et al. 2024; Radhakrishnan et al. 2024; Zhang et al. 2025). Additionally, BAP1 loss in tumor cells has been shown to alter the tumor immune microenvironment (Zhang and Wu 2023; Camp et al. 2024; Chang et al. 2024).

Despite these insights, the precise role of BAP1 in vivo in the liver and during the initial liver damage response is unknown. Given BAP1's association with cancer and the importance of PRC1/2 in fibrosis, uncovering the function of Bap1 early in tissue injury is critical for understanding how its loss contributes to later disease phenotypes. Previous work studied the combined deletion of Bap1 and Tp53 loss to understand cell fate changes in organoids but have not explored the role of BAP1 in vivo (Artegiani et al. 2019). Here, we generate a mouse hepatocyte-specific model of Bap1 loss to explore and characterize the changes that occur in liver biology in an in vivo system. Our work demonstrates that hepatocyte-specific deletion of Bap1 induces widespread changes to the immune environment and liver damage response during acute injury. Using high-resolution spatial analysis, we demonstrate changes to macrophage localization. Moreover, we show that Bap1 loss in the absence of damage induces an inflammatory response in the liver that is similar to the immune activation induced by damage. Together, these data suggest a complex interaction of all cells in the liver tissue milieu that are impacted by Bap1 loss in hepatocytes, including important immune cell populations that influence liver health, homeostasis, and tumorigenesis.

## Methods

### Mouse model

Cas9 Lox-Stop-Lox mice were purchased from Jackson Laboratory and bred in house (Strain #024857). The 16- to 20-week-old female mice were injected via the lateral tail vein with 1e12 viral particles of AAV8-U6 -sgRNA-TBG-Cre. On day, 10 mice were injected with either 1:9 dilution of CCl_4_ in olive oil or vehicle (olive oil) intraperitoneally at 10 µL/g bodyweight. Forty-eight hours after treatment, the left lobe was harvested for paraffin embedding and immunohistochemistry (IHC) and the right lobe was isolated for RNA. All animal experiments were approved by the Institutional Animal Care and Use Committee at The University of North Carolina at Chapel Hill.

### TIDE analysis

The efficiency of guide RNAs was tested by comparing the on-target editing to the off-targeted editing and to select strong guide for in vivo studies. On-target PCR primers for the in vitro test were BAP1-1 Forward CCACCACTGCCCTTTCAGAA Reverse CTGGTATCCACTCACGCTCC, gRNA sequence AGAATGGCCTCAGTGCAGTG. BAP1-2, which was selected for in vivo studies, Forward CCTGCCACACAGTTGGGTAT, Reverse CGGGCTGAAAGATCACCTCA, gRNA sequence TGAAGACGAGGATGACTACG. These were performed on both AML12 cells and mouse liver tissue from bulk RNA-seq experiments. Off-target sites were designed using Cas-OFFinder (Bae et al. 2014). Sequences and target sites are as follows, all coordinates are from mm10. Off-target 1 chr5:99,708,572 to 99,708,676 F TGGAGAAAGGGAAGGGGGAA, R GCAGCACAGATGGTTCCTTG, Off-target 2 chr5:36,484,712 to 36,484,879 F CAGTCGCTCGAGATCAAGGT, R TCGTTCTCGATCTGTCCCCT; Off-target 3 chr1:167,155,486 to 167,155,681 F TGTTTTGTTGCCACAGTGCT, R CCAGTTTCTGCACTGAATGGC. Off-target sites were tested on samples from liver tissue used in bulk RNA-seq. Products from these reactions were sequenced and then used as input for TIDE analysis at tide.nki.nl (Brinkman et al. 2014) One NTG sample was used as control for all other samples.

### Immunofluorescence

A 7 µM section of fresh-frozen liver tissue was sectioned on cryotome before fixing in fresh 4% paraformaldehyde in PBS. Sections were washed 3 times for 5 min at RT in PBS. Sections were blocked for 1 h at RT in PBS (5% Goat Serum, 0.3% Triton X-100). Sections were then washed 3 times for 5 min in PBS. Primary antibodies were diluted in antibody dilution buffer (1% BSA, 0.3% Triton X-100 in PBS) at the following dilutions GFP (1:100, Nacalai Tesque #04404-84 Lot M9N7092; Hnf4a #3113S Lot 2 1:400) added to slide and incubated at 4 °C overnight in a humidified chamber. Slides were washed 3 times for 5 min with PBS before incubation in secondary antibody in antibody dilution buffer for 1 h at RT (1:500 dilution, goat-anti rabbit or Alexafluor 647 or goat anti-rat Alexafluor 568, Invitrogen). Slides were washed 2 times for 5 min in PBS. DAPI was added at 0.5 µg/mL in PBS and incubated for 5 min at RT as the final wash. 1X TrueBlack was prepared in PBS from a 40× DMSO stock (Biotium #23011) and tissue was covered for 10 min. Slides were then rinsed 3 times in a staining jar with PBS and cover slipped using mounting media (Biotium #23001) before imaging on a Leica DMI8 inverted widefield microscope at 20×. Z-stack images were obtained and a multifocal image generated using the Leica LasX software. Final figure preparation was done using ImageJ to generate scale bars and inset regions.

### Immunohistochemistry

Livers from animals were excised and the left lobe was fixed in 10% neutral buffered formalin for 24 h at RT before paraffin embedding, slide sectioning, and hematoxylin and eosin (H&E) staining (UNC Pathology Services Core). For immunohistochemistry experiments, 5 μm liver sections were deparaffinized by incubating 3 times for 5 min at RT in CitriSolv and rehydrated by 2 sequential incubations for 10 min in 100% ethanol, 95% ethanol, and then followed by two, 5 min washes in dH_2_O. Antigen retrieval was performed in ImmunoRetriever Citrate unmasking solution in a TintoRetriever (Bio SB) on high pressure for 15 min. Sections were washed 3 times for 5 min in dH_2_O followed by incubating for 10 min in 3% hydrogen peroxide and finally washed in TBS with 0.1% Tween (TBST). Slides were incubated for 1 h at RT in TBST + 5% goat serum for blocking. Primary antibodies were diluted in TBST + 5% goat serum (Cre 1:100 Cell Signaling, 15036S Lot #2, F4/80 1:500 Cell Signaling catalogue 70076 Lot 9, CD3 1:400 Cell Signaling #78588 Lot 5, CPS1 1:400 Thermo PA5-106399 Lot ZK4547959) and incubated at 4° overnight in a humidified chamber. Slides were then washed 3 times for 5 min with TBST before addition of 1 to 3 drops of Signal Stain Boost Detection Reagent (Cell Signaling Technologies) directly to the slides and incubated for 30 min at RT. Slides were then washed 3 times for 5 min with TBST and 1 drop of Signal Stain DAB was diluted in 1 mL Signal Stain Diluent before adding to tissue for 5 min. Slides were immersed in H_2_O for 5 min twice and counterstained with hematoxylin for 1 to 2 min and then soaked in tap water for 1 to 2 min. Slides were then washed twice for 5 min in dH_2_O and then dehydrated by sequential incubations in 95% ethanol, 100% ethanol, and CitriSolv before mounting with Signal Stain Mounting Media. Slides were imaged on a Leica Dmi8 or an Olympus VS200 slide scanner.

### IHC quantification

IHC quantification was performed in QuPath (version 0.5.0) (Bankhead et al. 2017). Images were tiled, excluding tiles that were blurry or only within a vein. Positive cell detection was performed using the optical density sum in the QuPath module for Cre, F4/80, Cps1, and Cd3 and reported as percent positive. The *H*-score was calculated by multiplying the intensity by percent of cells positive—(0 × %neg) + (1 × %low) + (2 × %moderate) +(3 × %high). Six to seven equally sized regions per condition were selected for quantification (Bankhead et al. 2017). For central and portal veins, we focused on a smaller region of 500 by 500 pixel regions to restrict to specific vein type (*n* = 42 patches). Cutoffs for intensity were low = 0.05, medium = 0.4, high = 0.6 for F4/80 and low = 0.2, medium = 0.4, high = 0.6 for CD3. Score for the regions within each sample were plotted using ggplot2 (version 4.0.0) (Wickham 2016) and ANOVA was used to compute statistical significance between groups.

### Quantitative polymerase chain reaction

RNA was harvested with Monarch Total RNA extraction kit (NEB) and 1 µg of RNA was converted to cDNA using LunaScript RT by 25° for 2 min, 55° for 10 min, and 95° for 1 min. Two microliters of a 1:10 dilution of cDNA were then used as the input for qPCR with SsoFast (BioRad) with 300 nM gene-specific primers. Quantitation was performed as delta delta ct relative to Hprt and nontargeting control (Supplementary Table 1). BAP1 Forward GACCTTCAGAGTAAATGCCAGG, BAP1 Reverse ACCAACGTAGAAACCTTGCG, Tbp Forward GGGAGAATCATGGACCAGAA, TBP Reverse CCGTAAGGCATCATTGGACT.

### Histopathology review

All samples were examined using H&E–stained tissue sections with staining performed by the UNC Pathology Services Core. Histologic evaluation for zone 3 necrosis and steatosis was performed using a semi-quantitative scale by a board-certified pathologist with liver subspecialty training who was blinded to treatment assignments. Zone 3 necrosis was graded by the degree of injury and extent of involvement in zone 3. steatosis was graded by the degree of swollenness and injury.

### RNA-sequencing analysis

RNA was extracted from fresh-frozen liver samples (2 NTC Veh, 2 sgBap1 Veh, 3 NTC CCl_4_, 3 sgBap1 CCl_4_) using Applied Biosystems MagMAX mirVana Total RNA Isolation Kit (A27828). RNA sequencing libraries were generated from 500 to 1,000 ng input RNA using Illumina TruSEQ Stranded mRNA kit (Cat 20020594). Library concentration was calculated using Qubit 1× dsDNA HS Array. RNA sequencing libraries were sequenced 2 × 50 on an Illumina NextSeq200. Reads were aligned to the reference genome mm10 (GRCm38_p6) using STAR 2.7.6a and genes were quantified (Dobin et al. 2013) with Salmon 1.4.0 to gencode vM25 (Patro et al. 2017). Quality control was performed using SAMtools sort 1.10, Picard 2.22.4 CollectRnaSeqMetrics, calculate max read length, flagstat, and run FastQC 0.11.9 (Li et al. 2009; Andrews 2010).

Differential expression was performed with DESeq2 (version 1.42.1) using estimated counts from Salmon (Love et al. 2014; Patro et al. 2017). Following differential expression, shrinkage was applied to each coefficient using the lfcShrink function with type = “apeglm” (version 1.24.0) (Zhu et al. 2019; Supplementary Table 3). Results were visualized using ComplexHeatmap (version 2.18.0) and volcano plots with ggplot2 (Gu 2022; Wickham 2016).

Interaction between genotype and treatment were estimated with DESeq2 using the following design: “∼ treatment + genotype + treatment:genotype” (Love et al. 2014). Interaction terms were shrunk and genes with significant coefficients (adjust *P* < 0.05) were used for further analysis. Log normalized expression values were median centered across samples and clustered using 1-Pearson correlation distance and average linkage with the R package hclust (Supplementary Table 5).

### Gene set enrichment analysis

Shrunken log fold changes were used for downstream gene set enrichment analysis. The package msigdbr (version 7.5.1) was used to extract c5 and c8 gene sets from bulk data using the *GSEA* function from clusterProfiler (version 4.10.1) (Subramanian et al. 2005; Yu et al. 2012; Liberzon et al. 2015; Wu et al. 2021; Castanza et al. 2023; Dolgalev 2025). For enrichment/overrepresentation analyses, the “enricher” function was used. Gene sets were filtered by significance with a *P*-value threshold of 0.05, unless otherwise noted. Terms were grouped into “immune” and “non-immune” gene sets by keywords in Supplementary Table 6. These were visualized as waterfall plots using ggplot2 (Wickham 2016).

### Bap1 activity score

We used a previously defined transcriptional surrogate of BAP1 activity based on genes differentially expressed when BAP1 is altered (Sturgill et al. 2024). Using log_2_ normalized expression data, expression values for upregulated signature genes were multiplied by +1 and downregulated genes were multiplied by −1 and summed to derive a final score (Supplementary Table 7). Lower scores indicate less activity. We calculated the BAP1 activity score for a human liver cohort including both healthy and nonalcoholic steatohepatitis (NASH) (Chen et al. 2023). We obtained RNAseq fastq files from the Gene Expression Omnibus—GSE213621. Reads were aligned with STAR 2.7.11b to hg38 and genes were quantified using Salmon 1.10 with gencode v36 to obtain counts followed by BAP1 activity score as described above. Two outliers (GSM6590149 and GSM6589971) were removed for poor quality. Human genes in the BAP1 signature were converted to mouse orthologs using babelgene (version 22.9 https://igordot.github.io/babelgene/). Genes without a corresponding mouse ortholog were excluded (251/3,966 genes, 6%). We evaluated the sensitivity by removing these genes and recalculating BAP1 activity scores from the reduced gene set in the human cohort and found they were highly correlated (*R* = 0.98).

### Spatial transcriptomics

Spatial transcriptomics data were generated using the Molecular Cartography platform (Resolve Biosciences). The technology is derived from single-molecule fluorescent in situ hybridization (smFISH) and produces an approximate spatial resolution of 300 nm. Livers for 7 mice (1 NTC Veh, 1 sgBap1 Veh, 3 NTC CCl_4_, 2 sgBap1 CCl_4_) were harvested and immediately placed in O.C.T (TissueTek) before freezing in a dry ice/isopentane bath. Ten micrometer fresh-frozen liver tissue sections were cut on a cryotome and placed onto a Resolve Biosciences Molecular Cartography slide producing 4 NTC Veh, 2 sgBap1 Veh, 13 NTC CCl_4_, and 7 sgBap1 CCl_4_ ROIs. Samples were shipped to Resolve Biosciences for processing for data generation. A set of 100 targeted RNA probes were measured (Supplementary Table 8). Data were processed in the Resolve Biosciences online pipeline. Cell segmentation was achieved using Baysor and sample DAPI staining as a prior (Petukhov et al. 2022).

Putative cell types were assigned using robust cell type decomposition (RCTD) from the spacexr package (version 2.2.0) (Cable et al. 2022). The RCTD single-cell reference was built from liver cell type annotations and expression data from the mouse steady-state (StSt) liver cell atlas provided by Guilliams et al. (2022). Spatial cell types were called using default settings per-replicate by RCTD. Differences in cell type proportion across treatment conditions were determined using propeller (version 1.2.0) (Phipson et al. 2022) in “anova” mode with the transformation set to “logit.” To mediate spatial effects of sampling, we pooled all regions of interest per treatment group for analysis.

### Texture-based image analysis

F4/80 IHC slides were imported into R using OpenImageR (version 1.3.0) (Mouselimis et al. 2023). Images were converted to grayscale. For each image, a patch of 200 × 200 pixels of background were selected for background intensity normalization. The pixel intensity of each image was divided by the mean of its background pixels. A 1,500 × 1,500 pixel area was selected for analysis (Supplementary Table 9). Images were tiled into 8 × 8 grids (187.5 × 187.5 pixel) and filtered to remove tiles that encompassed mostly empty or veinous regions, using a Gaussian blurring effect from EBImage (version 4.48.0) and a threshold of more than 10% of pixels in a tile in top 10% of pixel values of 1,500 × 1,500 pixel image (Pau et al. 2010). Each tile was rasterized using raster (version 3.6-31) and aggregated by taking a 3 × 3 pixel average using the aggregate function from terra (version 1.8-15) (Hijmans et al. 2025; Hijmans et al. 2025). Filtered and aggregated tiles were run in gray-level co-occurrence matrices (glcm) algorithm (version 1.6.5) using contrast, homogeneity, entropy, second moment, variance, mean, and dissimilarity statistics across 0°, 45°, 90°, and 135° shifts with the number of gray levels (n_gray) set to 4 (Haralick et al. 1973; Zvoleff 2020). For each tile, the mean, standard deviation, quantile 1 and quantile 3, kurtosis, and skewness were calculated for each glcm metric with e1071 package (version 1.7-16) and used in prcomp (stats-package) for principal component analysis (PCA) analysis (Meyer et al. 2024). Tiles were visualized on the PCA using ggimage (version 0.3.4) (Yu and Xu 2025).

## Results

### BAP1 loss alters immune function

**Fig. 1.**
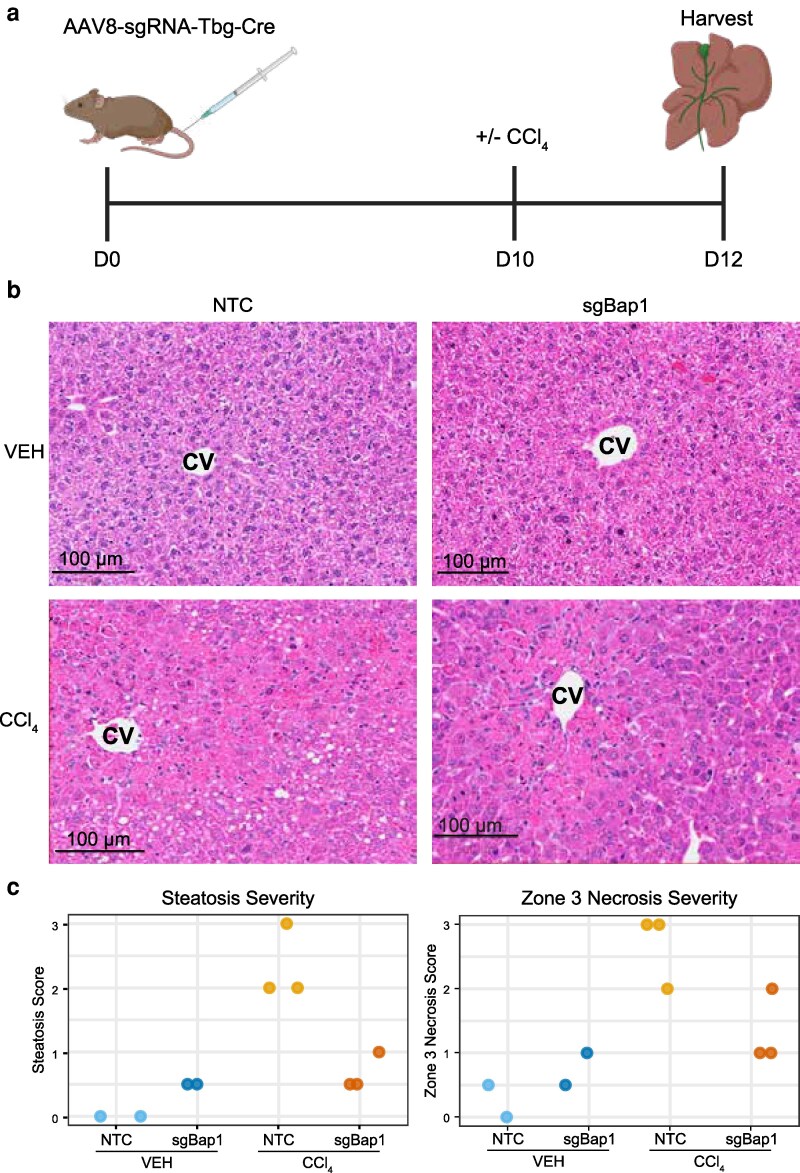
Histologic changes associated with Bap1 loss in hepatocytes. a) Schematic of experimental conditions and timeline. b) Representative H&E-stained images of all experimental conditions. NTC, non-targeting control; sgBap1, Bap1 targeting guide; VEH, vehicle treated; CCl_4_, carbon tetrachloride treated; CV, central vein. c) Steatosis and zone 3 necrosis grading. Images were graded by increasing severity from 0, +/− (0.5), + (1), ++ (2), to +++ (3). Panel A created in BioRender. Nenad, W. (2026) https://BioRender.com/kakp9vx

To understand how BAP1 contributes to the response to liver damage, we established a model of hepatocyte-specific depletion using CRISPR/CAS9. We tested 2 BAP1 guide RNAs (gRNAs) in the murine epithelial cell line AML12 by immunoblot to identify the strongest (Supplementary Fig. 1a to c). We then used this to generate a hepatocyte-specific AAV8-Tbg-Cre-sgRNA construct to evaluate the role of Bap1 in the liver and response to damage. This approach is commonly used to specifically target hepatocytes using a hepatocyte-specific thyroid hormone-binding globulin promoter (Tbg) and an AAV serotype (AAV8) that preferentially infects hepatocytes (Nakai et al. 2005; Yanger et al. 2013; Lee et al. 2020; Kiourtis et al. 2021) (Supplementary Fig. 1d). After 10 d, non-targeting control (NTC) and sgBap1 mice were treated with either carbon tetrachloride (CCl_4_) or vehicle and 48 h later livers were harvested (Fig. 1a). We observed robust IHC staining for Cre expression and transfection efficiency was comparable across all experimental groups with overall mean Cre positivity of 78.1% (ANOVA *P* = 0.327, Supplementary Fig. 1e and f). Reduction of Bap1 RNA expression in sgBap1 livers was confirmed by both qPCR and bulk RNA-seq (*t*-test *P* = 9e − 5 and Veh: NTC vs sgBap1 = 2.89 e − 7, CCl_4_: NTC vs sgBap1 = 5.73 e − 6, respectively, Supplementary Fig. 1g and h). Sanger sequencing of amplicons surrounding the BAP1 guide RNA sequence showed 30% to 40% editing efficiency compared with 3 off-target sites which were edited in only 0% to 5% of the sample (Supplementary Fig. 1i).

Histologic assessment of liver biopsy sections was performed by a board-certified liver pathologist (Fig. 1b and c; Supplementary Table 2). Steatosis was absent in normal NTC livers and upon CCl_4_ treatment was elevated to grade 2 or grade 3. In sgBap1 livers, cells appeared swollen. However, upon CCl_4_ treatment, steatosis was not elevated and few cells displayed fat droplets. Evaluation of zone 3 necrosis showed a similar patterning. Upon CCl_4_ treatment normal NTC livers displayed large necrotic areas with dead hepatocytes that had lost their nuclear staining and had recruitment of inflammatory cells. Vehicle-treated sgBAP1 livers had evidence of injury but without cells being completely necrotic yet but after CCl_4_ treatment, damaged areas were not elevated to the same extent as observed in the NTC livers. The zone 3 damage was further confirmed by the presence of the liver damage marker, Cps1, at damaged regions around central veins (Supplementary Fig. 2a and b).

### Transcriptional effect of Bap1 loss

**Fig. 2.**
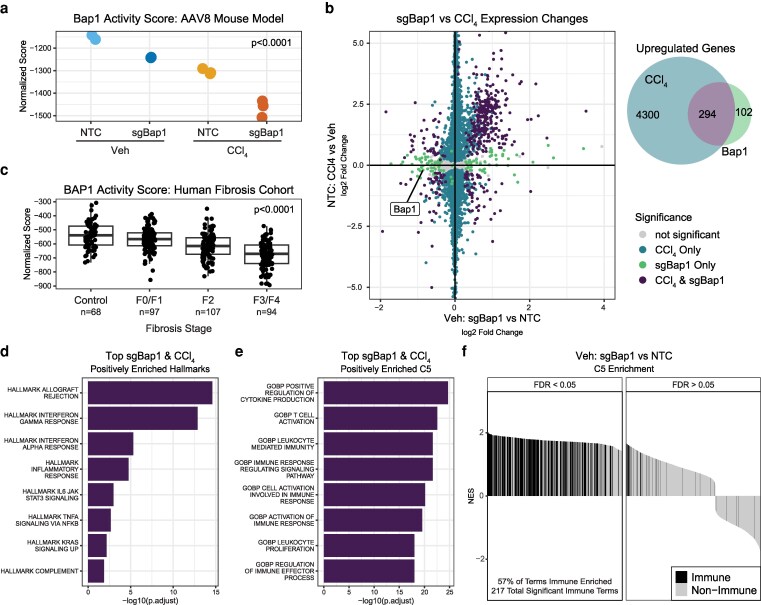
Transcriptional impact of Bap1 loss in liver. a) Bap1 activity score derived from bulk RNAseq across experimental mouse conditions. Statistical significance assessed by ANOVA test. b) Bap 1 activity score in a human fibrosis data set (GSE213621) stratified by fibrosis stage (68 control, 97 F0/F1, 107 F2, and 94 F3/F4). Statistical significance assessed by ANOVA test. c) Left, scatterplot of sgBap1-altered (Vehicle: sgBap1 vs NTC) and damage-altered (NTC: CCl_4_ vs Vehicle) bulk RNAseq log_2_ fold gene expression changes. Points are colored by significance level from DEseq2 (*P*adj < 0.5) and if shared between the two comparisons. Right, Venn diagram summarizing the overlap of upregulated genes between the two pairwise comparisons. Gene set enrichment analysis of shared sgBAP1 and CCl_4_ induced gene expression changes for d) Hallmarks and e) C5 gene sets. The top 8 enriched gene sets are shown ranked by –log_10_ (adjusted *P*-value). f) Waterfall plot of normalized enrichment scores (NES) from C5 GSEA performed on knockout specific (Veh: sgBap1 vs NTC) fold changes. Gene sets on the *x*-axis are ranked by decreasing NES. Black bars indicate gene sets containing immune related keywords.

We applied a previously defined transcriptional proxy of Bap1 pathway activity to our 4 mouse model conditions where lower scores reflect decreased BAP1 activity (Fig. 2a, see Methods) (Sturgill et al. 2024). We observed reduced BAP1 activity in sgBAP1 livers and in both CCl_4_-treated livers. We compared 2 pairwise differential expression analyses of bulk RNAseq to assess the similarity between CCl_4_-associated damage and hepatocyte-directed Bap1 loss: NTC-CCl_4_ vs NTC-Veh and sgBap1-Veh vs NTC-Veh (Fig. 2b). CCl_4_ elicited a strong transcriptional response (*n* = 8,721 genes, adjusted *P* < 0.05, Supplementary Table 3). Knockdown of Bap1 alone was associated with 551 genes differentially expressed compared to NTC in Veh-treated livers. We focused on the strong transcriptional upregulation of genes with Bap1 loss that overlapped with CCl4-induced genes (Fig. 2b, Venn diagram). Considering the slight elevation of histologic metrics of damage (Fig. 1c), we looked at BAP1 activity levels in an external human cohort of 368 healthy and nonalcoholic steatohepatitis liver samples (NASH) (Chen et al. 2023). We observed a similar decrease in Bap1 activity with increasing fibrosis stage (Fig. 2c). The shared up-regulated gene set displayed expression of immune-related pathways from Hallmark and C5 (Ontology) gene sets from MSigDB (Castanza et al. 2023; Fig. 2d and e; Supplementary Table 4). Returning to the full sgBap1-Veh vs NTC-Veh analysis, GSEA of the differentially expressed genes revealed that 57% of all significantly enriched C5 pathways were immune pathway–related (Fig. 2f). Together, these data suggest at the bulk liver expression level, strong immune cell expression signatures are induced by a hepatocyte-specific sgBap1 knockout similar to CCl_4_-induced damage.

### BAP1 loss alters the response to damage

**Fig. 3.**
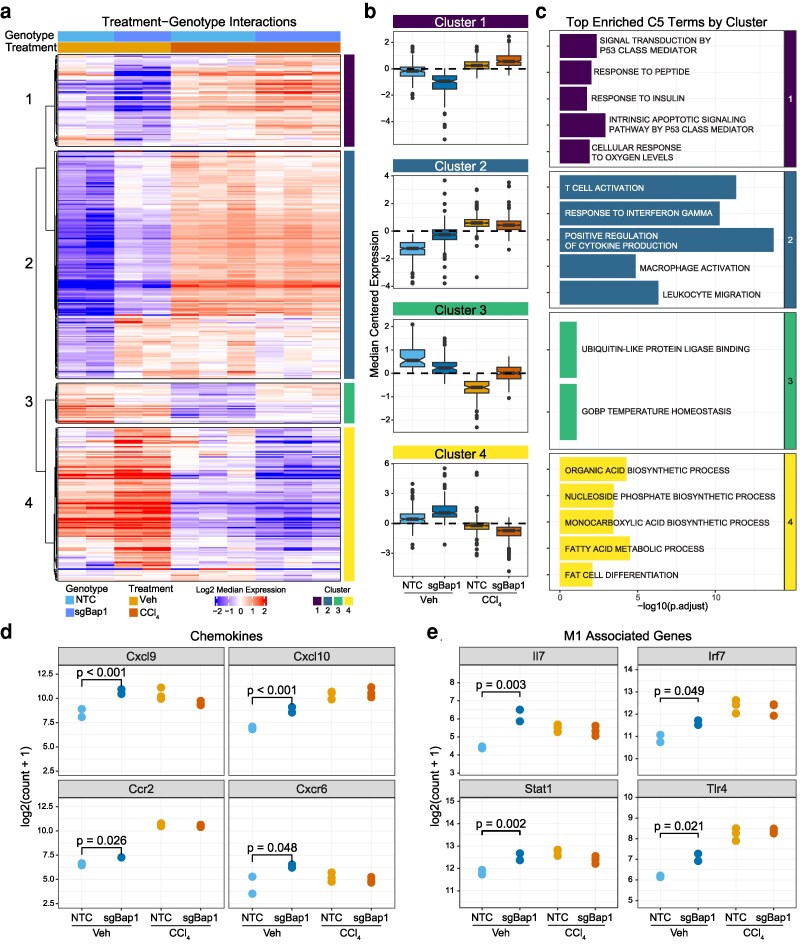
Bap1 alters transcriptional response to damage. a) Heatmap of median centered normalized Bulk RNAseq log-expression of genes with significant (*P* < 0.05) treatment-genotype interaction effects. Rows were hierarchically clustered using Pearson distance and average linkage and split into 4 clusters. b) Boxplots of median centered log-expression for genes in clusters identified in a). c) Top enriched MSigDB C5 ontology terms from clusters identified in A plotted by −log_10_ (adjusted *P*-value). d) Bulk RNAseq log_2_ normalized expression of chemokine and chemokine receptors. e) Bulk RNAseq log_2_ normalized expression of genes associated with M1 macrophage polarization.

We next evaluated how Bap1 knockout modulated the transcriptional response to liver damage. We identified 408 genes with a significant interaction between Bap1 loss and CCl_4_ treatment. Unsupervised clustering of these genes revealed 4 clusters with distinct patterns of expression (Fig. 3a).

Cluster 1 patterns showed genes with an overall upregulation of expression with CCl_4_ treatment but were markedly lower in sgBap1 vehicle-treated livers (Fig. 3b). Cluster 1 was strongly enriched for genes in signal transduction, cell-cycle regulation, and stress responses including genes such as Cdkn1a, Egr1, Mdm2, Myc. Consistent with this, we observed an increase in ki67-positive hepatocytes after damage that was significantly reduced by Bap1 loss (*χ*^2^ with Yates correction *P* < 0.001) (Supplementary Fig. 2c).

Cluster 2 was defined by an immune-activated expression phenotype in sgBap1-Veh that was elevated compared with NTC but lower than the levels observed with CCl_4_ treatment. We observed marked enrichment for signatures related to cytokine signaling, adaptive and innate immune responses, interferon gamma pathway, and tumor necrosis factor pathway (Fig. 3c; Supplementary Table 5). We identified several chemokines associated with immune recruitment in the liver (Fig. 3d). For example, Cxcl9 and Cxcl10 are expressed by hepatocytes and recruit T cells (Marra and Tacke 2014; Cao et al. 2021). Ccr2 is a receptor associated with M1-polarized macrophages and monocyte infiltration and Cxcr6 is a natural killer T-cell receptor that responds to signals from Kupffer and sinusoidal cells (Marra and Tacke 2014; Cao et al. 2021). Vehicle-treated sgBap1 livers had elevated expression of genes characteristic of a pro-inflammatory M1 macrophage phenotype. For example, Il7, Irf7, Stat1, and Tlr4 were upregulated to levels comparable with those observed in CCl_4_ treatment (Fig. 3e). However, there was no similar increase in anti-inflammatory M2 macrophage genes Fn1, Msr1, Tgfbr2, or Stat6 (Supplementary Fig. 3). While we cannot conclusively state that macrophages are the source cell for this signature, these data suggest that Bap1 loss potentiates a pro-inflammatory environment in livers that potentially involves the activation and recruitment of M1 macrophages.

Cluster 3 was enriched by a small number of transcriptional regulators, including the critical hepatocyte transcriptional regulators Cebpb and Foxo1, which play a role in lipid and glucose metabolism (Wang et al. 2005; Tikhanovich et al. 2013). The expression level of these genes was reduced with damage and sgBap1 relative to the NTC. Taken with cluster 1, these results suggest that Bap1 loss impairs hepatocyte maintenance and transcriptional regulation similar to observed changes with liver damage.

Cluster 4 was characterized by genes with higher expression in vehicle-treated sgBap1 mice. This cluster was enriched with terms related to fatty acid, nucleoside, and organic acid metabolism. These terms were defined by genes known to regulate and alter the lipid profile of the liver. One example, Srebf1, a transcription factor that controls hepatic lipogenesis, is upregulated in the context of NAFLD/MASLD (Im et al. 2011; Li et al. 2023). Furthermore, downstream targets of Srebf1 such as Acly and Elovl6 were upregulated, both of which are involved in lipogenesis (Supplementary Table 3; Horton et al. 2003).

These data demonstrate the loss of Bap1 impacts the transcriptional response to damage. Key pathways such as cell-cycle regulation, lipogenesis, and inflammation were all altered by the loss of Bap1. Immune cell signatures were dominant the changes, therefore, we further looked to see if these gene signature shifts were associated with altered cell type composition of the liver.

### BAP1 loss alters the cell composition of the liver

**Fig. 4.**
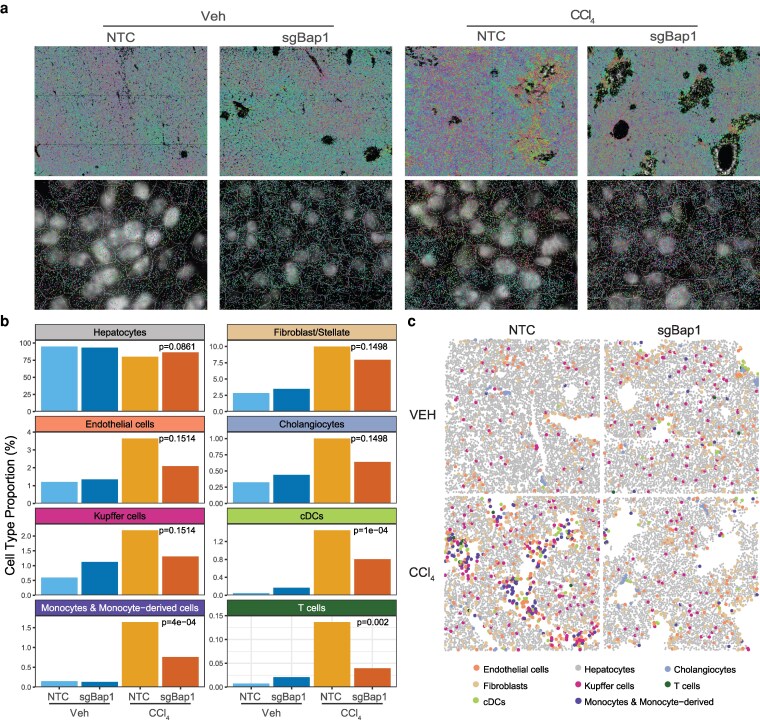
Bap1 loss in hepatocytes alters cell populations and recruitment. a) Representative images from Resolve Biosciences spatial transcriptomics for all experimental conditions. Top, visualization of 100 smFISH probes; Bottom, cell segmentation overlayed on DAPI stained images with smFISH probes. b) Proportion of putative cell types per experimental condition. Spatial transcriptomics data was segmented to a cell level and cell type labels were annotated with RCTD. Variation in cell populations was assessed by ANOVA. c) Representative reimaged spatial patterning of segmented cells colored by annotated cell type.

To further characterize the changes in cell type composition, we performed subcellular spatial transcriptomics on another cohort of 7 livers for 100 genes associated with hepatic cell types, differentiation, and proliferation on the Resolve Biosciences platform (Fig. 4a; Supplementary Table 8). After cell segmentation, we annotated cell types using a liver cell atlas (Meyer et al. 2024). Of the 17 cell types in the atlas, we were able to confidently call 8 cell types with at least 100 cells per type (Fig. 4b). sgBap1 livers had an increase in Kupffer cells, conventional dendritic cells (cDCs), and T cells (Fig. 4b). Following damage, the increase of Kupffer cells, monocyte-derived cells, cDCs, and T-cells observed in NTC livers was blunted by sgBap1. The cell type labels were applied back to the *xy* coordinates to evaluate spatial patterns (Fig. 4c). The increase in immune cells observed in sgBap1 maintained a uniformly distributed pattern. However, upon damage, sgBap1 did not show the same influx of immune cells to damaged areas as the sgNTC livers did, despite similar levels of Cps1 expression and ki67-positive cell numbers increased (Supplementary Fig. 2a to c).

**Fig. 5.**
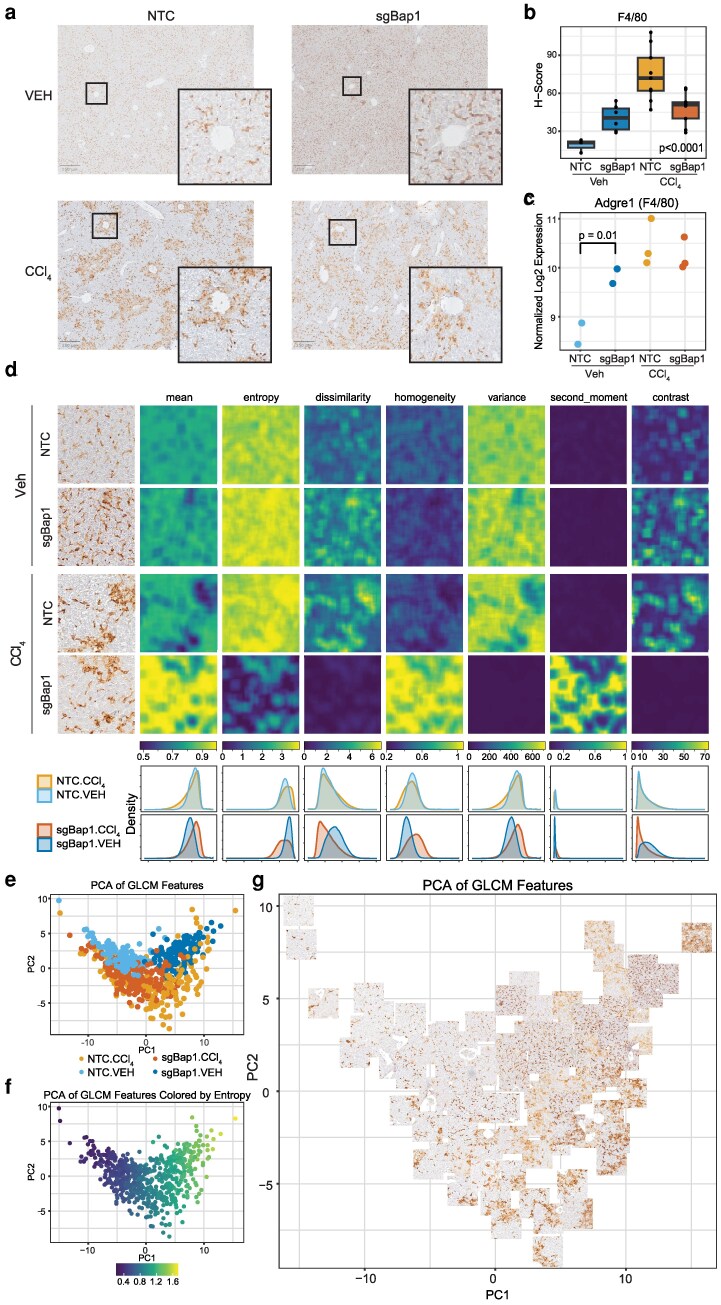
Altered macrophage spatial patterning with Bap1 loss. a) Representative F4/80-stained images of all experimental conditions. Image inlay of magnified area around a central vein. b) *H*-score of F4/80 immunohistochemical stain. Statistical significance was assessed with one way ANOVA. c) Bulk RNAseq log_2_ normalized gene expression of Adgre1 (F4/80). d) Image-based texture analysis using gray level co-occurrence matrix analysis (GLCM) on F4/80 IHC images. Representative tiles of all 4 experimental conditions for 7 GLCM features—mean, entropy, dissimilarity, homogeneity, variance, second moment, and contrast. Bottom, distributions of all pixel values for each filter colored by condition. Principal component analysis (PCA) of tile level GLCM summary statistics colored by e) condition, f) GLCM entropy value, or g) subset of tile-level representations.

Given that Kupffer cells, the liver's resident macrophage, were prominently altered in both the spatial and bulk RNA data, we sought to validate these findings histologically using the macrophage marker F4/80. We observed similar trends, showing an increased staining and density of F4/80 in sgBap1 livers but still retained a dispersed spatial patterning as in the NTC livers (Fig. 5a). After damage, NTC livers drastically increased staining for F4/80 and the cells clustered near damage around central veins with staining appearing more diffuse (Fig. 5a). However, in sgBap1 livers, the response to damage was less robust with less intense staining and reduced recruitment to areas of damage. The visual changes were confirmed with the semi-quantitative *H*-score, which was increased in sgBap1 livers with Veh, but displayed a muted and altered response with CCl_4_ treatment (Fig. 5b). These data were consistent with bulk RNA-seq expression levels for Adgre1, the gene encoding F4/80 (Fig. 5c). We also noted minor differences in T cells (CD3+ cells) with a slight increase in IHC staining in both sgBap1 conditions (Supplementary Fig. 4c).

We used image-based texture analysis (GLCM) to statistically quantify and summarize spatial patterns of F4/80 staining and other visible changes in tissue architecture (Haralick et al. 1973; Zvoleff 2020). We analyzed 7 GLCM pixel level texture features (Supplementary Fig. 5) and summarized at a tile level. These features quantify how pixel intensities are distributed and related across an image, capturing overall signal intensity (mean), variability (variance), the degree of pixel intensity differences (contrast and dissimilarity), the uniformity of pixels (homogeneity and angular second moment), and the randomness or complexity of texture (entropy). These features identified differences both with sgBap1 and CCl_4_ treatment (Fig. 5d). NTC liver tiles had high entropy and variance features defining the dispersion of F4/80 stain cells across tissues. In sgBap1, we noted increased entropy, dissimilarity, and contrast and decreased homogeneity and variance suggesting a shift toward a more complex, heterogeneous, and less uniform texture (Fig. 5d). With CCl_4_ damage, sgBap1 tissues showed dramatic differences in texture patterns for features. We plotted summary metrics of each feature for each tile using PCA and found separation of image tiles by both sgBap1 and CCl_4_ status (Fig. 5e; Supplementary Table 10). The PCA loadings provided insights into key features driving the clustering (Supplementary Figs. 6 and 7; Supplementary Table 11). The first PC was driven by features indicative of cell number, while PC2 was driven by features of variation (Fig. 5f; Supplementary Fig. 6). For a more interpretable visualization, we plotted a subset of the points using their tile image (Fig. 5g). While the sgBap1 tiles show clear increase in F4/80 stain similar to treated samples, upon damage sgBap1 tissues show altered macrophage recruitment with lower diffuseness. We evaluated the location of the F4/80 stain aggregation by estimating the *H*-score at local regions around central veins (zone 3) and portal veins (zone 1). The F4/80 enrichment was specific to the central vein where CCl_4_ predominantly causes damage (*P* = 0.00046) with no differences observed surrounding portal veins (*P* = 0.49; Supplementary Fig. 8). Together, these data suggest that a modest increase in macrophages occurs in undamaged sgBap1 livers, but that macrophage recruitment to zone 3 damage is altered by Bap1 loss in hepatocytes. Moreover, this analysis highlights the impact of quantitative assessment of spatial patterns which can reveal more detailed insights into how loss of Bap1 impacts the spatial distribution of macrophages.

## Discussion

Germline loss of BAP1 leads to a tumor predisposition syndrome and somatic mutations of BAP1 are common in primary liver cancer, uveal melanoma, and mesothelioma (Harbour et al. 2010; Rai et al. 2016; Cheung et al. 2017; Sturgill et al. 2024). However, few studies have interrogated the function of BAP1 outside of cancer. We sought to define the role of BAP1 in normal liver cells and to determine if these changes contribute to a role for Bap1 as a potential early driver for liver disease and later cancer risk. The activation of hepatic stellate cells by damaged hepatocytes is a key initial driver of the liver damage response, and dysfunction of this interaction can lead to maladaptive repair and fibrosis (Seki et al. 2015; Kamm and McCommis et al. 2022). Therefore, we investigated Bap1's role in normal hepatocytes and how it contributes in response to acute liver damage. We depleted Bap1 specifically in hepatocytes using CRISPR/CAS9 which led to increased inflammation and altered recruitment of immune cells in the uninjured liver.

These data suggest that Bap1 depletion in hepatocytes on its own activates an inflammatory response that mimics changes seen after liver injury. Notably, similar “pre-injured” states were found in the histology data with changes to macrophage localization and steatosis. The link between Bap1 loss and an immune response has been observed in several prior studies (Figueiredo et al. 2020; Zhang and Wu 2023; Camp et al. 2024; Chang et al. 2024; Liang et al. 2024; Radhakrishnan et al. 2024; Zhang et al. 2025). For example, in renal cell carcinoma, putative loss of function mutations in BAP1 are associated with increased chromatin accessibility at genes associated with inflammatory response and nuclear factor kappa-light-chain-enhancer of activated B cells (NF-kB) signaling and in colorectal cancer cell lines, loss of BAP1 lead to increased chemokine expression associated with immune cell recruitment (Camp et al. 2024; Chang et al. 2024). However, Bap1 loss does not lead to a consistent directionality of that immune response across different tissues. For example, in uveal melanoma, loss of BAP1 leads to an immunosuppressive environment by disrupting NF-kB signaling, while in mesothelioma BAP1, loss is associated with interferon alpha and gamma and in increase inflammatory response (Figueiredo et al. 2020; Zhang and Wu 2023). Our data support Bap1 loss as an inducer of an inflammatory response in the liver and many of these inflammatory signatures were also upregulated in normal livers in response to damage. We noted an increase in chemokine expression in sgBap1 livers which are potentially acting as recruiters for the increased immune cells we observed. There was also increased expression of genes involved in the inflammasome potentially reflecting a primed proinflammatory milieu driven by Bap1 loss in hepatocytes. However, the gene expression changes we observed in bulk RNA-seq data could be due to direct changes in hepatocytes or effects from other cells in the liver or changes in proportion of those cells.

Paradoxically, the transcriptional and immune cell response to damage of Bap1-depleted livers was blunted compared to wild-type livers. We noted this reduced immune response in both the expression data and in the histology data. CCl_4_-treated sgBap1 livers had reduced zone 3 necrosis and reduced migration of F4/80 macrophages to damage at the central vein. Recent work has shown that during damage, zone 3 necrotic regions are predominantly populated and cleared by infiltrating monocyte-derived macrophages while Kupffer cells predominately stay in zones 1 and 2 (Feng et al. 2025). Even though inflammatory pathways were already partially activated with Bap1 loss, there is a disruption of signaling of immune response to damage. This could be due to changes in how cytokine signals are sent or received by adjacent cells or could reflect an exhaustion of the liver environment driven by the premature activation of an immune response in Bap1-depleted hepatocytes or delayed necrosis. Further studies examining the timeline of repair and injury in the context of BAP-loss are needed to distinguish these possibilities.

While we do not know the direct mechanism through which hepatocytes influence immune cell recruitment and response, the increase in expression of chemokines in uninjured Bap1-depleted livers, which notably do not become further elevated with damage, suggests a dysfunction cross-talk mechanism. Additional studies evaluating the cell-type specific functions and the involvement of Bap1 in hepatocytes and other cell types during the response to injury are needed to understand how this inter-cell communication impacts the immune response. Together, these experiments suggest an early role for Bap1 in priming the liver for maladaptive repair and support Bap1 loss as an early event that contributes to later liver disease; an idea supported by human fibrosis data where BAP1 activity was reduced with increasing severity of fibrosis. Our experiments implicate Bap1 having a broader role in maintaining homeostasis in the uninjured liver. Future studies will need to carefully dissect the temporal roles of Bap1 and its molecular functions to maintain proper liver function and response to injury.

## Supplementary Material

jkag047_Supplementary_Data

## Data Availability

All processed expression data are available in supplementary tables. Bulk RNA sequencing data is available on GEO at GSE310239. Single-cell spatial data and imaging data are available on synapse at syn72116265 and FigShare (https://doi.org/10.25387/g3.31337344). All software used in this work is described in the Methods section and scripts are deposited at https://github.com/hoadley-lab/bap1-spatial. sgBAP1 and sgNTC plasmids derived from these are available upon request. Supplemental material available at G3 online.
