# Supplementary material for: Hepatocyte-targeted Bap1 reduction in the liver primes an inflammatory transcriptional response
Source: G3 (Bethesda). 2026 Feb 19;16(5):jkag047. doi: 10.1093/g3journal/jkag047 (PMC13148380; doi:10.1093/g3journal/jkag047)
Supplement: jkag047_Supplementary_Data [file jkag047_supplementary_data.zip › Supplemental_Material_G3-2026-406585.docx]

**Supplemental Figures and Table Descriptions**

**
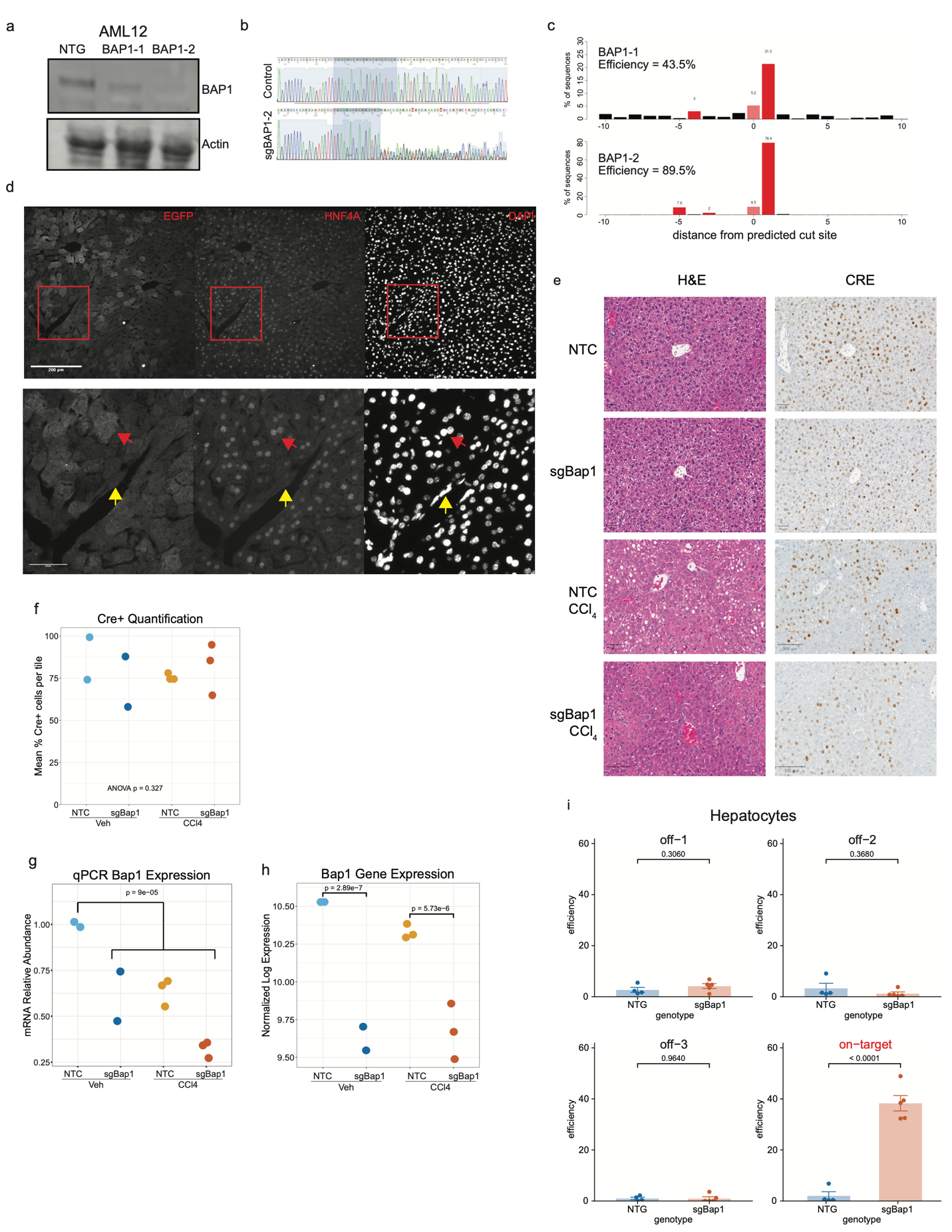
**

**Supplemental Figure 1. Histologic and Expression quantification of Cre and Bap1.** A) Immunoblot of Bap1 or Actin from AML12 cells transduced with sgNTG or one of two sgBAP1 gRNAs. b) Example Sanger sequence trace from stronger guide in panel A depicting region around predicted cut site. c). Efficiency of editing for two Bap1 guide RNAs from TIDE analysis. d) Immunofluorescence imaging for EGFP, Hnf4a, or DAPI at 20x (top row) or the highlighted inset region (red box; bottom row). Yellow arrow depicts example of cells that lack GFP and Hnf4a, red arrow depicts GFP/Hnf4a double positive nuclei. e) Representative Hematoxylin and eosin (H&E)-stained and immunohistochemistry stained Cre images. f) Quantification of immunohistochemistry of Cre+ cells g) qPCR of Bap1 expression levels. h) Bulk RNA-seq gene expression levels of Bap1. i) On and off-target editing efficiencies from liver tissue used in bulk RNA-seq experiments.


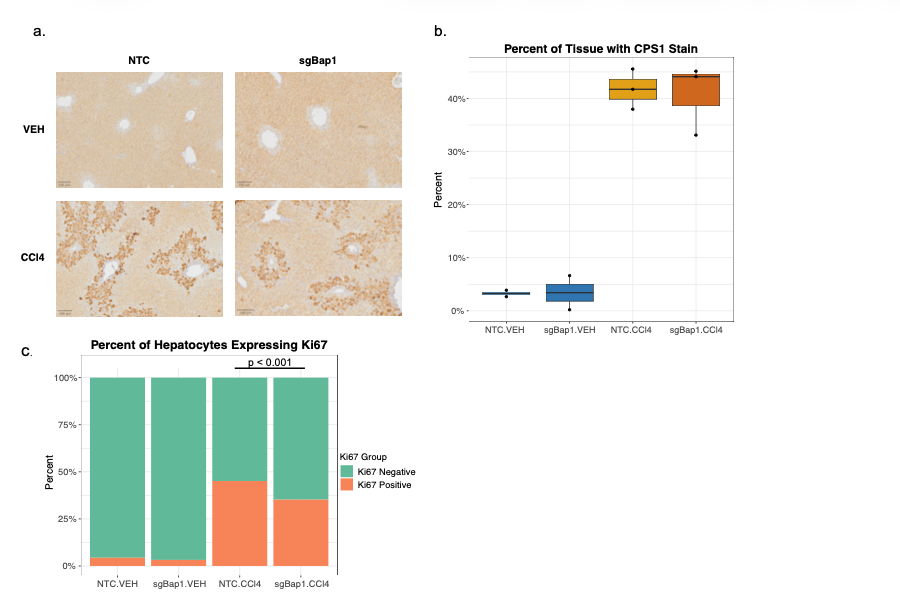


**Supplemental Figure 2. Comparison of damage extent and proliferation.** a.) Immunohistochemistry of Cps1 stained livers. b.) Percent of cell stained with Cps1 quantified from images in a. c.) Percent of hepatocytes expressing Ki67 from Resolve Spatial Transcriptomics data.


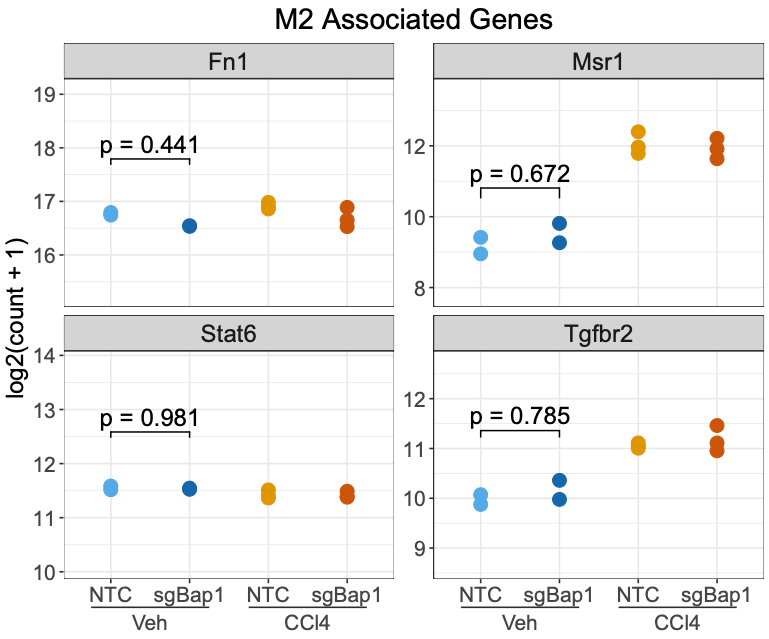


**Supplemental Figure 3. Pro-inflammatory M2 macrophage gene expression.** Log2 normalized expression of genes associated with M2 macrophage polarization.


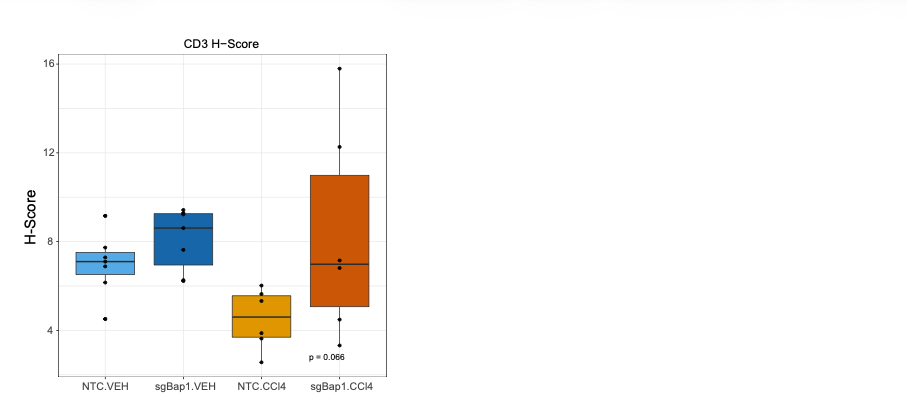


**Supplemental Figure 4. H-Score of Cd3 cells**. H-score of Cd3 immunohistochemical stain around central veins. Statistical significance is assessed with one way ANOVA (n = 26 patches).


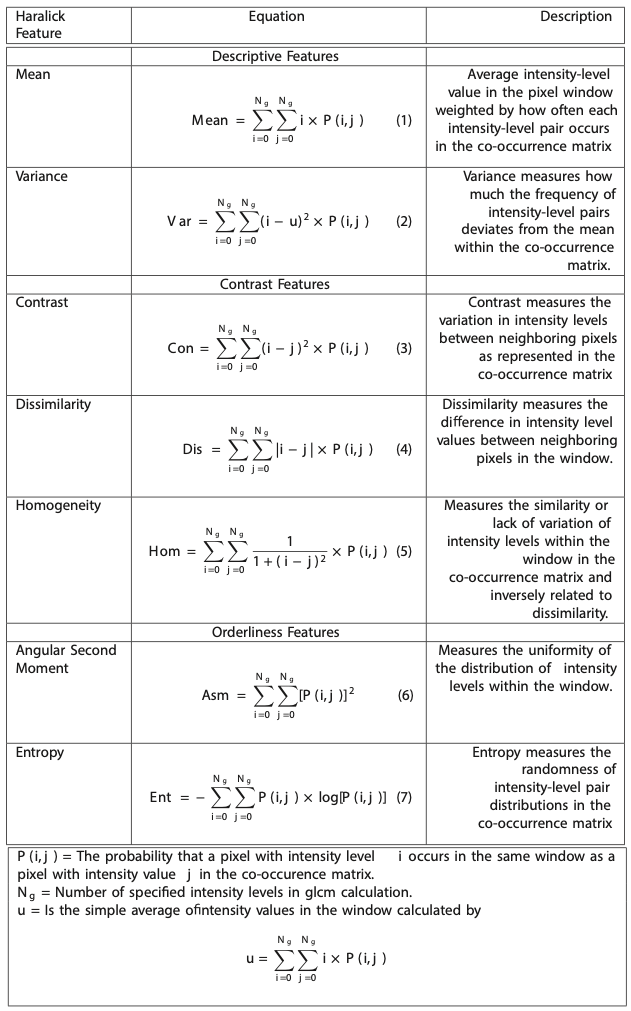


**Supplemental Fig 5. Image-based texture analysis.** Equations and descriptions for Haralick features performed in GLCM texture analysis Fig 5D-G (Haralick et al. 1973; Zvoleff 2020).


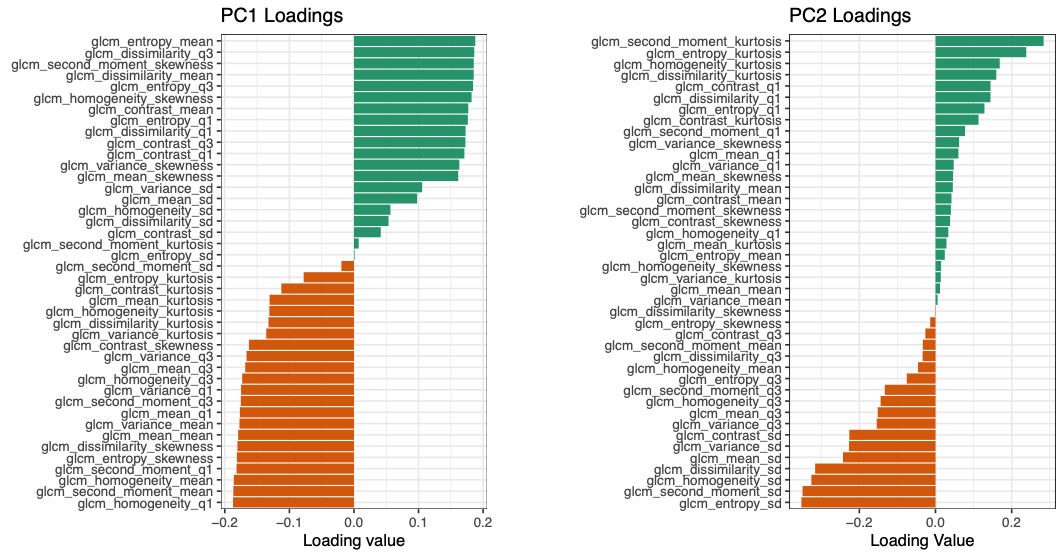


**Supplemental Figure 6. Waterfall plot of PCA loading values for both PC1 and PC2.**


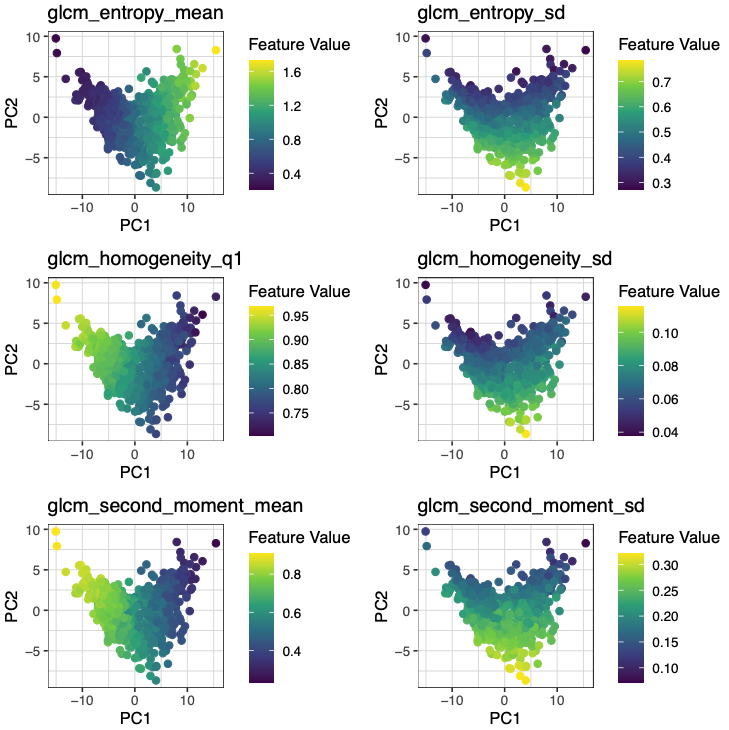


**Supplemental Figure 7 PCA of GLCM texture values colored by top 3 PC loadings from PC1 (left) and PC2 (right).**

**
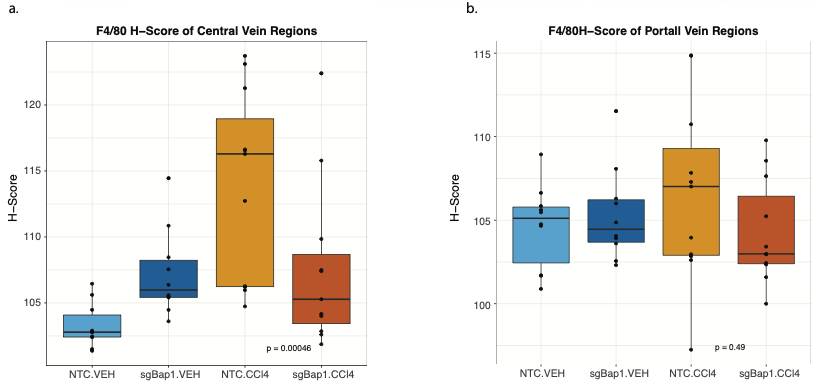
**

**Supplemental Figure 8. Comparison of central vein and portal vein F4/80 expression.** a.) H-scores of regions surrounding the central vein (n = 42 patches). b.) H-Scores of regions surrounding the central vein (n = 42 patches).

**Supplemental Tables**

1. qPCR results for BAP1.
2. Pathological summary of H&E slides for Necrosis and Steatosis related to Figure 1C.
3. Shrunken log2Fold changes of all genes. Each tab is a specific comparison noted below
   1. sgbap1.Veh vs NTC
   2. SgBap1.CCl4 vs NTC
   3. NTC.CCl4 vs VEH
   4. sgBap1.CCl4 vs Veh
4. GSEA of shared significantly enriched genes from Figure 2B. Tab for each analysis noted below.
   1. Hallmark pathways Figure 2D.
   2. C5 pathways Figure 2 E,F.
5. GSEA of genes with significant interaction effects. Tab for each gene set noted below
   1. Interaction genes that had significant change and their cluster in Figure 3A.
   2. C5 gene set enrichment output of clusters from interaction terms Figure 3C.
6. Immune Terms identified from C5 MSigDB data set for Figure 2F.
7. Bap1 activity scores of samples. Tab for each data set noted below.
   1. Bap1 activity score of fibrosis dataset.
   2. Bap1 activity score of hepatic Bap1 KO dataset.
8. Gene Probes Selected for Spatial Transcriptomics in Resolve Biosciences.
9. F4/80 images and their reference patches as well as tissue patches from Figure 5D-G.
10. PC values from GLCM analysis with treatment groupings from Figure 5D-G.
11. PC loading values from GLCM analysis with treatment groupings from Figure 5D-G
